# Supplementation of highly concentrated β-cryptoxanthin in a satsuma mandarin beverage improves adipocytokine profiles in obese Japanese women

**DOI:** 10.1186/1476-511X-11-52

**Published:** 2012-05-14

**Authors:** Masako Iwamoto, Katsumi Imai, Hideaki Ohta, Bungo Shirouchi, Masao Sato

**Affiliations:** 1 Graduate School of Health and Nutrition Sciences, Nakamura Gakuen University, 5-7-1 Befu, Jounan-Ku, Fukuoka 814-0198, Japan; 2 Laboratory of Nutrition Chemistry, Department of Bioscience and Biotechnology, Faculty of Agriculture, Graduate School, Kyushu University, 6-10-1 Hakozaki, Higashi-ku, Fukuoka 812-8581, Japan

**Keywords:** β-cryptoxanthin supplementation, Serum HMW-adiponectin, Serum PAI-1, Intervention study, Satsuma mandarin

## Abstract

**Background:**

Serum β-cryptoxanthin levels are lower in overweight subjects than in normal subjects. Abnormalities of adipocytokine profiles in obesity subjects have been reported. There are several reports that serum β-cryptoxanthin levels in them were relatively lower than normal subjects.

**Objective:**

We hypothesize that supplementation of highly concentrated β-cryptoxanthin improves serum adipocytokine profiles in obese subjects. This study tested the association between β-cryptoxanthin intake and serum adipocytokine levels.

**Methods:**

An intervention study consisted of a 3-week long before-and-after controlled trial, where β-cryptoxanthin (4.7 mg/day) was given to 17 moderately obese postmenopausal women.

**Results:**

The results indicated no significant changes in body weight or body mass index (BMI). Serum β-cryptoxanthin levels increased significantly by 4-fold. Serum high molecular weight (HMW)-adiponectin levels increased significantly, while serum plasminogen activator inhibitor (PAI)-1 levels decreased.

**Conclusions:**

We concluded that increasing the intake of β-cryptoxanthin to approximately 4 mg per day for 3 weeks may have beneficial effects on the serum adipocytokine status and consequently alleviate progression of metabolic syndrome.

## Introduction

**Figure 1:**
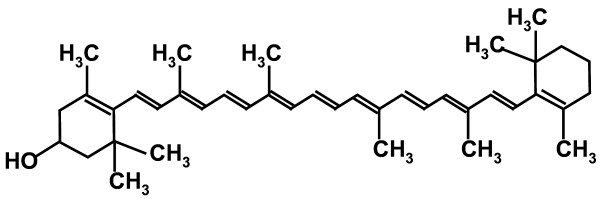
The chemical structure of β-cryptoxanthin.

Serum β-cryptoxanthin levels (Figure 1) are lower in overweight subjects than in normal subjects, irrespective of dietary intake [1]. In a case-controlled study, which was part of a larger survey of 2895 subjects with body mass indexes (BMI) greater than 25, serum β-cryptoxanthin levels were found to be lower than in subjects with BMIs lower than 25 [2]. Adipose tissue store excess energy in the form of fat and secretes physiologically active substances known as adipocytokines [3]. Among these compounds, adiponectin is the most abundant adipose-specific protein. Plasma adiponectin levels are negatively correlated with BMI in men and women [4]. Growing evidence suggests that high-molecular weight (HMW)-adiponectin is the most physiologically active form present in metabolic disorders [5]. Plasminogen activator inhibitor-1 (PAI-1) is a non-specific adipocytokine and its levels are positively correlated with the area of visceral fat determined by computed tomography (CT) scan of obese and non-obese subjects [6].

Serum β-cryptoxanthin levels have been reported in subjects with metabolic syndrome [1], but no intervention studies have provided dietary β-cryptoxanthin to subjects. We hypothesize that supplementation of highly concentrated β-cryptoxanthin improves serum adipocytokine profiles in obese subjects. This study aimed to assess the degree of adiposity and serum adipocytokine levels in middle-aged postmenopausal women with a BMI of 25–30, who had been administered a beverage containing high concentrations of β-cryptoxanthin for 3 weeks.

## Methods and materials

### Subjects

Seventeen postmenopausal obese women participated in this study. Obesity was defined as a body mass index (BMI) greater than 25 kg/m^2^, according to the Japan Society for the Study of Obesity. Criteria for participation in this study included no past history of diabetes mellitus, no use of prescribed medicine, and no history of smoking. This study was approved by the Nakamura Gakuen University Committee (No. 05–009), in accordance with the Declaration of Helsinki. Written informed consent was obtained from all participants.

The daily nutrient intake of the subjects was monitored for 3 days before the start and 3 days after the study by means of dietary records. Subjects were instructed to continue with their usual diets, but were prohibited from the intake of citrus fruits, persimmon, and the juices of either fruits during the period of the investigation. The nutrient intake was calculated using Excel Eiyou-kun ver. 3 (Kenpaku-sha, Tokyo, Japan). The intervention study was a 3-week long before-and-after controlled trial, where 200 ml of a beverage containing β-cryptoxanthin (1.56 mg/serving and 4.7 mg/day) was provided to the subjects. Other ingredients of the beverage which we determined were as follows, energy (27 kcal/serving), water (183.4 g/serving), protein (0.6 g/serving), lipids (< 0.2 g/serving), ash (< 0.2 g/serving), total carbohydrate (6.1 g/serving), sugars (3.78 g/serving), dietary fiber (1.1 g/serving), potassium (71.6 mg/serving), calcium (12.0 mg/serving), sodium (0.8 mg/serving), magnesium (5.7 mg/serving), vitamin C (49.10 mg/serving), β-carotene (0.19 mg/serving). The beverage was prepared from residues generated by the process used in the manufacture of satsuma mandarin juice. The residues contained higher concentrations of β-cryptoxanthin than the manufactured juice. The beverage was prepared by resuspending the residues in water, and β-cryptoxanthin levels in the beverage were identical in each serving.

### Blood sampling and biochemical measurements

Blood samples were assayed for total cholesterol, triglyceride, high-density lipoprotein (HDL) cholesterol, glucose, HMW-adiponectin, leptin, total PAI-1, and insulin in a commercial laboratory (SRL, Fukuoka, Japan). Plasma carotenoid levels were measured in a commercial laboratory (BIKEN, Kyoto, Japan). Plasma carotenoids, including, α -carotene, β-carotene, β-cryptoxanthin, lycopene, and lutein-zeaxanthin, were extracted and concentrated using standard methods. These compounds were then quantified by reversed-phase high-performance liquid chromatography (HPLC).

### Statistical analyses

All values are expressed as mean ± SE. The statistical difference was determined using a two-tailed paired *t*-test. *P* < 0.05 was considered significant.

## Results

The baseline characteristics of the subjects at the beginning of the investigation are shown in Table 1. Postmenopausal women were selected on the basis of BMI. The highest BMI was 32.1 and the lowest was 23.1. There were no differences in body weight or BMI after the treatment. Serum triglyceride levels at the end of the study tended to decrease (*P* = 0.057). There were no significant differences in total serum low-density lipoprotein (LDL) and HDL cholesterol levels. Subjects had blood pressure measurements in the normal range at the beginning of the study, and this was also the case for the serum parameters (Table 2). After drinking the beverage containing β-cryptoxanthin for 3 weeks, serum β-cryptoxanthin levels significantly increased 4-fold. The results show that serum HMW-adiponectin levels at the end of the study were significantly higher compared with the initial period. Serum total PAI-1 levels at the end of the study tended to be lower (*P* = 0.052). The levels of other carotenoids, such as lycopene, α-carotene, β-carotene, and zeaxanthin, were not significantly different after the treatment.

**Table 1:** Comparison of clinical data and daily intake of nutrients before and after 3-weeks intake of a β-cryptoxanthin beverage (n = 17)

**Characteristics**	**Before**	**After**	**Change**^**1)**^	** *P* -value**
Age (years)	55 ± 1.7	55 ± 1.7		
Height (cm)	154.5 ± 0.9	154.5 ± 0.9		
Body weight (kg)	64.9 ± 1.7	64.8 ± 1.7	−0.1	0.473
BMI (kg/cm^2^)	27.1 ± 0.6	27.1 ± 0.6	0	0.722
Blood pressure				
SBP (mmHg)	134 ± 4.6	128 ± 3.7	−6	0.052
DBP (mmHg)	80 ± 2.2	79 ± 2.1	−1	0.366
Dietary intake per day^2)^				
Energy (kcal)	1659 ± 101	1676 ± 96	17	0.805
Protein (g)	69.3 ± 3.7	67.5 ± 3.5	−1.8	0.597
Fat (g)	51.4 ± 3.8	50.9 ± 3.6	−0.4	0.943
Carbohydrate (g)	225.2 ± 15.4	223.9 ± 15.6	−1.4	0.916
Dietary fibre (g)	16.5 ± 1.0	20.1 ± 1.6	3.6	0.035
β-Cryptoxanthin (mg)	723 ± 237	4719 ± 6	3996	0.000
Vitamin C (mg)	133 ± 15	231 ± 7	98	0.000

Data show means  ±  S.E.

Abbreviations: BMI, body mass index; SBP, systolic blood pressure; DBP, diastolic blood pressure.

^1)^ Change implies subtraction of ‘before’ data from ‘after’ data.

^2)^ Included beverage.

**Table 2:** Comparison of FBG, serum lipid, adipocytokine, and carotenoid levels before and after 3-weeks intake of a β-cryptoxanthin beverage (n = 17)

**Characteristics**	**Before**	**After**	Change*	** *P* -value**
FBG (mg/dl)	101 ± 3	101 ± 2	1	0.749
Total-cholesterol (mg/dl)	216 ± 6	217 ± 6	1	0.827
LDL-cholesterol (mg/dl)	135 ± 6	136 ± 6	0	0.949
HDL-cholesterol (mg/dl)	64 ± 3	64 ± 3	0	0.803
Triglyceride (mg/dl)	116 ± 14	100 ± 12	−16	0.057
Leptin (mg/dl)	15.7 ± 1.8	15.5 ± 2.0	−0.2	0.853
HMW-Adiponectin (mg/ml)	9.8 ± 1.2	11.1 ± 1.1	1.3	0.009
Total PAI-1 (ng/ml)	32 ± 3	28 ± 3	−4	0.052
β-cryptoxanthin (mg/ml)	0.28 ± 0.04	1.15 ± 0.08	0.86	0.000
Lycopene (mg/ml)	0.18 ± 0.02	0.18 ± 0.01	0.00	0.741
α-carotene (mg/ml)	0.09 ± 0.02	0.09 ± 0.01	0.00	0.795
β-carotene (mg/ml)	0.37 ± 0.04	0.38 ± 0.03	0.00	0.793
Zeaxanthin (mg/ml)	0.07 ± 0.01	0.07 ± 0.01	0.00	0.901

Data show means ± S. E.

Abbreviations: FBG, fasting blood glucose; HMW, high molecular weight; PAI-1, plasminogen activator inhibitor-1* Change implies subtraction of ‘before’ data from ‘after’ data.

## Discussion

This study investigated the potential effects of β-cryptoxanthin supplementation on obesity. There were no significant effects on body weight and BMI after the 3-week treatment. However, β-cryptoxanthin supplementation improved the adipocytokine status, as indicated by increased serum HMW-adiponectin levels and decreased PAI-1 levels.

A previous study [7] reported that a Mediterranean diet leads to increased serum adiponectin levels in metabolic syndrome subjects. In a short-term study, Shimada *et al.*[8] reported that supplementation of the diet of 17 elderly men with normal BMI values with Oolong tea (1,000 ml/day) for 4 weeks increased the plasma adiponectin levels, but without any decrease in body weight. Sluijs *et al.*[9] reported that higher intake of dietary carotenoid, particularly β-cryptoxanthin and lycopene, was associated with a reduced prevalence of metabolic syndrome in middle-aged elderly men. Daily β-cryptoxanthin intakes in the Sluijs *et al.*[9] study were 1/37^th^ of that found in the current study. However, the dietary β-carotene and lycopene intake in the Sluijs *et al.*[9] study was almost the same as that found in the current study. Thus, daily consumption of milligram-scale carotenoids, including, β-carotene, lycopene, and β-cryptoxanthin, might alleviate metabolic syndrome.

Jones *et al*. [10] reported a correlation between serum PAI-1 levels and carotenoid bioavailability in an intervention study, where fish-oil esters of plant sterols were added to the diets of moderately overweight and hypercholesterolemic subjects over a period of 4 weeks. Compared with supplementation with safflower oil esters, supplementation with fish-oil esters of plant sterols significantly elevated the serum levels of retinol and β-carotenes and decreased serum PAI-1 levels. Thus, an increased in serum carotenoid levels might result in a decrease of serum PAI-1 levels. In this study, serum β-cryptoxanthin levels increased, whereas PAI-1 levels decreased.

In conclusion, increasing β-cryptoxanthin intake to approximately 4 mg per day for 3 weeks could have beneficial effects on serum adipocytokine status, and subsequently alleviate progression of metabolic syndrome. Further case–control study in larger scale may be needed to prove sound relationships between β-cryptoxanthin intake and the risk of development of metabolic syndrome.

## Abbreviations

HMW-adiponectin: High molecular weight adiponectin; PAI-1: Plasminogen activator inhibitor; BMI: Body mass index; DBP: Diastolic blood pressure; FBG: Fasting blood glucose; SBP: Systolic blood pressure.

## Competing interests

The authors declare that they have no competing interests.

## Authors’ contributions

MI conceived the study, participated in the design of the study, acquired data, performed statistical analysis and drafted the manuscript. KI and HO carried out the survey and organized the data. BS and MS participated in the design on the study and helped conducting the study. All authors read and approved the final manuscript.

## Funding

This work was in part supported by a grant from the Research and Development Program for New Bio-industry initiatives of the Bio-oriented Technology Research Advancement Institution, Japan.
